# The relationship between a microfinance-based healthcare delivery platform, health insurance coverage, health screenings, and disease management in rural Western Kenya

**DOI:** 10.1186/s12913-020-05712-6

**Published:** 2020-09-14

**Authors:** Molly Rosenberg, James Akiruga Amisi, Daria Szkwarko, Dan N. Tran, Becky Genberg, Maya Luetke, Sina Kianersi, Jane Namae, Jeremiah Laktabai, Sonak Pastakia

**Affiliations:** 1 Department of Epidemiology and Biostatistics, Indiana University School of Public Health, 1025 E. 7th Street, Bloomington, Indiana USA; 2 Department of Family Medicine, Moi University School of Medicine, Eldoret, Kenya; 3 Department of Family Medicine, Moi University School of Medicine, PO Box 4606 30100, Eldoret, Kenya; 4 Moi Teaching and Referral Hospital, Eldoret, Kenya; 5 Department of Family Medicine and Community Health, The University of Massachusetts Medical School, Worcester, MA USA; 6 Department of Family Medicine, Warren Alpert School of Medicine, Brown University, Providence, RI USA; 7 Purdue Kenya Partnership, Purdue University College of Pharmacy, Eldoret, Kenya; 8 Department of Epidemiology, Johns Hopkins Bloomberg School of Public Health, Baltimore, MD USA; 9 Department of Epidemiology and Biostatistics, School of Public Health, College of Health Sciences, Moi University, Eldoret, Kenya; 10 Webuye Health and Demographic Surveillance System, Moi University, Eldoret, Kenya

**Keywords:** Microfinance, Health insurance, Health screening, Kenya

## Abstract

**Background:**

Structural barriers often prevent rural Kenyans from receiving healthcare and diagnostic testing. The Bridging Income Generation through grouP Integrated Care (BIGPIC) Family intervention facilitates microfinance groups, provides health screenings and treatment, and delivers education about health insurance coverage to address some of these barriers. This study evaluated the association between participation in BIGPIC microfinance groups and health screening/disease management outcomes.

**Methods:**

From November 2018 to March 2019, we interviewed a sample of 300 members of two rural communities in Western Kenya, 100 of whom were BIGPIC microfinance members. We queried participants about their experiences with health screening and disease management for HIV, diabetes, hypertension, tuberculosis, and cervical cancer. We used log-binomial regression models to estimate the association between microfinance membership and each health outcome, adjusting for key covariates.

**Results:**

Microfinance members were more likely to be screened for most of the health conditions we queried, including those provided by BIGPIC [e.g. diabetes: aPR (95% CI): 3.46 (2.60, 4.60)] and those not provided [e.g. cervical cancer: aPR (95% CI): 2.43 (1.21, 4.86)]. Microfinance membership was not significantly associated with health insurance uptake and disease management outcomes.

**Conclusions:**

In rural Kenya, a microfinance program integrated with healthcare delivery may be effective at increasing health screening. Interventions designed to thoughtfully and sustainably address structural barriers to healthcare will be critical to improving the health of those living in low-resource settings.

## Background

Low- and middle-income countries (LMICs) are generally characterized by higher mortality rates and lower life expectancy compared to high-income countries [1]. Kenya is no exception with an average life expectancy of 63 years, placing it in the bottom quartile of all countries [2]. This high mortality is often attributed to a high burden of infectious diseases [3–6]. Overall, 5.6% of the Kenyan population is living with HIV, [7] with a disproportionate burden among women [2, 8]. Kenya also ranks among the 14 countries with the highest burden of TB, multidrug-resistant TB, and TB/HIV co-infection [9]. Non-communicable disease (NCDs) also pose a growing health threat [6, 10, 11]. In 2014, NCDs accounted for 27% of all deaths in the country [12, 13]. Nearly a third of Kenyans have high blood pressure, and diabetes is rapidly increasing in the country [12, 14].

Despite the high burden of infectious diseases and increasing burden of NCDs in Kenya, healthcare service utilization remains low [15]. One significant barrier to healthcare consistently identified across LMICs is poverty. Poverty can suppress healthcare access through inability to afford services, low service availability, and geographic accessibility issues [16, 17]. In Kenya, poverty has been associated with less healthcare utilization [18, 19] and more than a third of the overall population was living below the poverty line in 2016 [20]. Additionally, the Kenyan health system is largely funded by out-of-pocket payments from patients and efforts to address the inequity arising from this structure have been mostly unsuccessful [21]. Thus, poverty is a key barrier to uptake of health screening, and the treatment and management of health conditions [22].

Universal health coverage, or the ability of all people to obtain the quality health services they need without suffering financial hardship, [23] may provide an effective pathway toward expanding healthcare access. In 2005, WHO member countries, of which Kenya is a member, passed a resolution urging countries to adopt universal health coverage and, in 2010, the government of Kenya stated that health is a universal right [24, 25]. Yet, little progress has been made toward universal health coverage in Kenya and only about 10–20% of the population has health insurance [3, 21, 26]. The National Health Insurance Fund (NHIF) is the primary source of health insurance in the country and may represent a key turning point for improving healthcare access [21, 27]. The package of NHIF benefits was recently expanded to allow beneficiaries to receive comprehensive inpatient and outpatient care in both the public and private sectors [27]. The government-supported insurance is offered at a low cost, providing affordable premium payment requirements for low-income populations. Despite these improvements, barriers to coverage remain, particularly for rural, informally employed citizens who tend to have the lowest rates of participation in NHIF [21, 28].

**Fig. 1:**
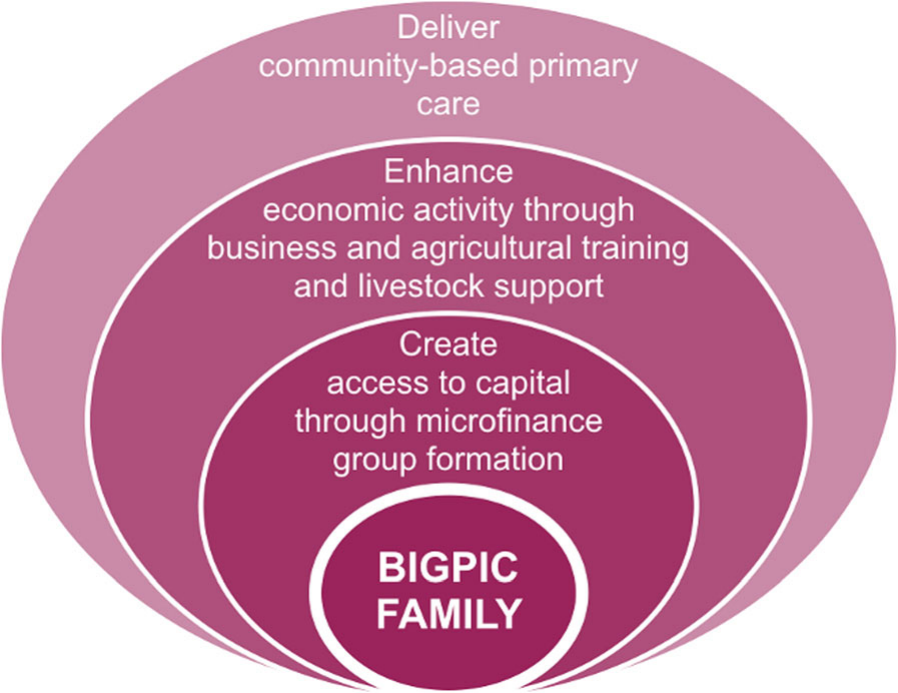
Conceptual model of the BIGPIC Family intervention components

In rural Western Kenya, where this study took place, the Bridging Income Generation with GrouP Integrated Care (BIGPIC) Family program aims to address some of these structural barriers to healthcare in order to improve screening and healthcare access for local residents [29]. BIGPIC Family offers a combination of interventions designed to synergistically improve health and well-being, including: group-based microfinance services, education about NHIF, point-of-care screening and management for hypertension and diabetes, group-based primary care delivery during microfinance meetings, business literacy, and agricultural training (Fig. 1) [29–31]. The microfinance groups are the platform through which much of the BIGPIC programming and healthcare is delivered.

We evaluated the associations between participation in BIGPIC Family microfinance groups and (1) NHIF coverage, (2) health screenings, and (3) disease management outcomes. We hypothesized that participation in the BIGPIC Family microfinance groups would have several positive health-related outcomes, including higher uptake of NHIF, higher uptake of disease screenings, and improved disease management among those with a chronic disease diagnosis.

## Methods

### Study population and setting

This cross-sectional study was conducted in two communities of similar size (~ 15,000 population) and demographics in rural, western Kenya. These two communities were chosen based on the level of their engagement with AMPATH’s BIGPIC program at the time of the study. AMPATH is a partnership between Moi University, Moi Teaching and Referral Hospital, North American universities led by Indiana University, and the Kenyan Government with a mission of teaching, care and research. BIGPIC began implementation in Community 1 in 2016 but had not yet rolled out to Community 2 at the time of our data collection.

We drew our sample from three different study populations. First, we sampled 100 BIGPIC Family microfinance group members, by targeting the full rosters of 7 groups randomly selected from all groups with at least 6 months of active membership in Community 1. Second, we identified a sample of 100 people in Community 1. Third, we identified a sample of 100 people in Community 2. Both community samples were randomly sampled from an enumeration of all community residents developed for a recent hypertension study in the area [32]. Only participants age 18 years and above were eligible. Participants were not eligible to be interviewed twice if they happened to be sampled for both the microfinance group and community sample in Community 1. An a priori power calculation was conducted for our desired sample size of *n* = 300. For common outcomes (at 50% prevalence), we maintained at least 80% power to detect all prevalence ratios greater than 1.3. With less common outcomes (at 10% prevalence), the effect sizes necessary to maintain 80% power increased up to 2.2. Thus, we were well-powered to detect even small effects with common outcomes, but underpowered to detect small effects with uncommon outcomes.

The microfinance groups are the platform through which much of the BIGPIC programming is delivered. Individual members meet regularly to take out and repay group-funded loans, and they may choose to receive the full complement of other available interventions, including: 1) screening and care for diabetes and hypertension and 2) NHIF education. Some intervention spillover to non-microfinance community members is expected if they engaged in health screening or NHIF training at community-wide events.

### Data collection

Between November 2018–February 2019, trained local fieldworkers collected survey data from 300 participants. All data were self-reported using a tablet-based, quantitative survey administered in the local language of Kibukusu with REDCap software. The survey covered a range of topics including socio-demographic information, microfinance group experience, and health screenings (see survey instrument in Supplemental Material). All interviews were conducted at home in a private area, after obtaining informed consent. Ethical approval was provided by the Indiana University Institutional Review Board (#1705661852) and the Moi University Institutional Research and Ethics Committee (#00030702).

### Key variables

BIGPIC Family microfinance group membership was the primary exposure. Participants who self-reported being a current member of a BIGPIC Family microfinance group with a start date of at least 6 months prior to the interview date were considered exposed. As a sensitivity analysis, we also considered duration of participation as a continuous variable, based on the difference between start date and interview date.

The unexposed group was composed of non-microfinance group members of both Community 1 (the community in which the program had already rolled out) and Community 2 (the community in which the program had not yet rolled out). To evaluate whether to combine these communities for analysis, we assessed whether there were differences between them with respect to our key health outcomes and socio-demographic characteristics. We found no statistical differences between the two communities for our key outcomes, and no differences in socio-demographic characteristics with the exception of slightly different wealth index distributions (Supplemental Table 1).

We operationalized NHIF health insurance coverage as a potential outcome of BIGPIC Family group membership. Although NHIF education was provided through BIGPIC microfinance groups, the groups have not historically been used to directly enroll participants. Participants were asked to self-report if they had active NHIF coverage, which was confirmed with SMS messages to an NHIF information number.

As our primary health outcomes, we queried if participants had ever been screened for each of the following health conditions: HIV, diabetes, hypertension, tuberculosis, and cervical cancer. BIGPIC Family routinely provides screening and care for some conditions (diabetes and hypertension), but not others (HIV, tuberculosis, cervical cancer). We also assessed two disease management outcomes among the small subset of participants who reported a diagnosis of hypertension, diabetes, or HIV (*n* = 32): 1) current medication for condition, and 2) healthcare visit to manage condition within the last 6 months.

We also collected sociodemographic data on: sex, age, marital status, educational attainment, employment outside the home, and socio-economic status (SES). SES was operationalized by querying household assets. We calculated a weighted index of ownership of 20 items, then categorized participants in quartiles, aligned with the methodology of the 2014 Kenyan DHS) [33].

### Statistical analysis

To understand the relationship between microfinance group membership and each of the health insurance, health screening, and disease management outcomes, we used log-binomial models to estimate prevalence ratios. We compared unadjusted results to results adjusted for age, sex, and marital status. To explore potential differences by sex and by SES, we ran models stratified by sex and by household assets dichotomized at the median. In the case of ‘0’ cell counts in the stratified analyses, we added 0.5 to each cell. To assess whether there were statistical differences in effect sizes by sex and SES, we introduced an interaction term between each of sex and household assets with microfinance group membership. Interaction terms with Wald *p*-values < 0.05 indicated statistically significant differences.

Since people who select into microfinance groups are likely to have different personality profiles compared to those that do not and this may also correlate with engagement in health screenings and care, we conducted a sensitivity analysis examining the relationships of interest among a study population restricted to only include microfinance group members. We then used duration of group membership as a proxy for the exposure, with a cutpoint dividing short-term members (< 12 months) from long-term members (greater than or equal to 12 months). We compared the prevalence of our health outcomes between short and long-term members to assess whether the results were of similar magnitude to our main findings.

## Results

Overall, 300 men and women enrolled in the study. We targeted 422 potential participants for interview, 21 were ineligible, 2 refused, and we were unable to locate 99 for a response rate among those eligible to participate of 75%. Temporary outmigration for school or employment accounted for most (62%) of those we were unable to locate. A majority of the participants were women (63%), were currently married (73%), and did not work outside the home in the last 30 days (80%) (Table 1). Participants ranged in age from 18 to 96 (median age: 40) and lived in households with, on average, 5 people. One-third of participants were BIGPIC Family microfinance group members. Members were more likely to be women, older, and currently married. There were no significant differences by microfinance membership for educational attainment, formal employment, household assets, and household size.

**Table 1:** Characteristics of the study population of 300 residents of two communities in rural western Kenya, 2018–2019

		Microfinance group membership^a^		
	Total *N* = 300	Yes *N* = 100	No *N* = 200	P*
***Socio-demographic characteristics***				
	N (%)	N (%)	N (%)	
**Gender**				< 0.0001
Male	112 (37.3)	19 (19.0)	93 (46.5)	
Female	188 (62.7)	81 (81.0)	107 (46.5)	
**Age**				0.0006
< 20	24 (8.0)	1 (1.0)	23 (11.5)	
20–29	51 (17.0)	11 (11.0)	40 (20.0)	
30–39	68 (22.7)	32 (32.0)	36 (18.0)	
40–49	76 (25.3)	30 (30.0)	46 (23.0)	
50+	81 (27.0)	26 (26.0)	55 (27.5)	
**Marital status**				< 0.0001
Never married	45 (15.1)	1 (1.0)	44 (22.1)	
Currently married	219 (73.2)	84 (84.0)	135 (67.8)	
Divorced/separated	35 (11.7)	15 (15.0)	20 (10.1)	
Missing	1	0	1	
**Education**				0.2
None/ Some primary	92 (30.7)	31 (31.0)	61 (30.5)	
Primary	116 (38.7)	45 (45.0)	71 (35.5)	
Secondary	69 (23.0)	20 (20.0)	49 (24.5)	
Post-secondary	23 (7.7)	4 (4.0)	19 (9.5)	
**Work outside home (last 30 days)**				0.4
Yes	59 (19.7)	17 (17.0)	42 (21.1)	
No	240 (80.3)	83 (83.0)	157 (78.9)	
Missing	1	0	1	
**Household asset quartile**^b^				0.7
Q1	70 (25.6)	21 (23.6)	49 (26.5)	
Q2	70 (25.6)	27 (30.3)	43 (23.2)	
Q3	61 (22.3)	19 (21.4)	32 (22.7)	
Q4	73 (26.6)	22 (24.7)	51 (27.6)	
Missing	26	11	15	
**Current NHIF coverage**				0.2
Yes	33 (11.0)	14 (14.1)	19 (9.5)	
No	266 (89.0)	85 (85.9)	181 (90.5)	
Missing	1	1	0	
**Household size [Mean (SD)]**	5.0 (2.27)	5.2 (2.37)	4.8 (2.21)	0.2
*Health screening characteristics*				
**HIV screening**				< 0.0001
Yes	252 (84.0)	96 (96.0)	156 (78.0)	
No	48 (16.0)	4 (4.0)	44 (22.0)	
**Diabetes screening**				< 0.0001
Yes	124 (41.3)	77 (77.0)	47 (23.5)	
No	176 (58.7)	23 (23.0)	153 (76.5)	
**Hypertension screening**				< 0.0001
Yes	191 (63.7)	96 (96.0)	95 (47.5)	
No	109 (36.3)	4 (4.0)	105 (52.5)	
**Tuberculosis screening**				0.0001
Yes	31 (10.3)	20 (20.0)	11 (5.5)	
No	269 (89.7)	80 (80.0)	189 (94.5)	
**Cervical cancer screening**				0.005
Yes	36 (19.2)	23 (28.4)	13 (12.2)	
No	152 (80.9)	58 (71.6)	94 (87.9)	
*Disease management outcomes, among those who report HIV, diabetes, or hypertension diagnoses (n = 32)*				
**Medical visit in last 6 months**				0.1
Yes	23 (71.9)	10 (90.9)	13 (61.9)	
No	9 (28.1)	1 (9.1)	8 (38.1)	
**Currently taking medication**				0.1
Yes	17 (53.1)	8 (72.7)	9 (42.9)	
No	15 (46.9)	3 (27.3)	12 (57.1)	

*p-value reported for chi-square test for categorical variables and t-test for continuous variables. *P*-values calculated among observations with non-missing values

^a^Member of BIGPIC Family microfinance group for at least 6 months prior to interview

^b^Measured by adding up the self-reported value (at time of purchase) of 20 key items in participant’s household

Overall, 11% of all participants had active NHIF health insurance coverage. Health screening rates varied by condition: HIV (84%), diabetes (41%), hypertension (64%), tuberculosis (10%), and cervical cancer (19% - calculated among women only). Among those who reported a diagnosis with HIV, diabetes, or hypertension (*n* = 32), nearly three-quarters (72%) reported a medical visit in the last 6 months. Just over half reported being currently on medication for their condition (53%).

Health insurance coverage among microfinance group members was 14% compared to 10% in non-members; however, we did not observe a statistically significant association [aPR (95% CI): 1.26 (0.64, 2.27)] (Table 2). There were strong associations between microfinance group membership and health screening. Microfinance members were over three times as likely to report diabetes screening [aPR (95% CI): 3.46 (2.60, 4.60)], about twice as likely to report hypertension screening [aPR (95% CI): 1.96 (1.56, 2.46)], over three times as likely to report tuberculosis screening [aPR (95% CI): 3.31 (1.56, 7.03)]. Among women, microfinance group members were over twice as likely to report cervical cancer screening [aPR (95% CI): 2.43 (1.21, 4.86)]. For each of these outcomes, the unadjusted results were similar in magnitude to the results adjusted for age, gender, and marital status. HIV screening was also higher among microfinance members (96%) compared to non-members (78%); however, we did not observe statistically significant associations between microfinance membership and HIV screening [aPR (95% CI): 1.11 (0.64, 2.47)].

**Table 2:** Relationship between microfinance group membership, health screening, and disease management, among 300 residents of two communities in rural western Kenya, 2018–2019

	Unadjusted			Adjusted^a^		
	PR (95% CI)		*p*	aPR (95% CI)		*p*
Current NHIF coverage	1.49	(0.78, 2.84)	0.2	1.26	(0.64, 2.47)	0.5
*Health screening outcomes*						
HIV screening	1.23	(1.13, 1.34)	< 0.0001	1.11	(0.96, 1.29)	0.2
Diabetes screening	3.28	(2.50, 4.30)	< 0.0001	3.46	(2.60, 4.60)	< 0.0001
Hypertension screening	2.02	(1.74, 2.35)	< 0.0001	1.96	(1.56, 2.46)	< 0.0001
Tuberculosis screening	3.64	(1.81, 7.29)	0.0003	3.31	(1.56, 7.03)	0.002
Cervical cancer screening	2.42	(1.27, 4.64)	0.008	2.43	(1.21, 4.86)	0.01
*Disease management outcomes, among those diagnosed with HIV, diabetes, or hypertension (n = 32)*						
Medical visit in last 6 months	1.47	(1.00, 2.16)	0.05	1.20	(0.68, 2.10)	0.5
Currently taking medication	1.70	(0.92, 3.13)	0.1	1.30	(0.55, 3.08)	0.5

^a^Adjusted for age (categorized at above/below age 40 years), marital status (categorized as currently married vs not), and gender. As cervical cancer screening rates were only calculated among female participants, the adjusted results for this outcome were not adjusted for gender

Among those who reported diagnoses with HIV, diabetes, or hypertension (*n* = 32), the associations between microfinance membership and disease management outcomes were not statistically significant (Table 2). The prevalence ratios for the relationship between microfinance group membership and both disease management outcomes were above the null, but small in magnitude [Medical visits within the last 6 months: aPR (95% CI): 1.20 (0.68, 2.10); Reporting current medication for their health condition: aPR (95% CI): 1.30 (0.55, 3.08)]. Due to the small sample size of this sub-group, our ability to precisely measure these associations was limited.

The relationship between BIGPIC microfinance membership and the tuberculosis screening outcome was stronger in men compared to women (Table 3, Wald *p*-value = 0.05). We observed no statistical difference between men and women for the relationships between microfinance and the other health screening and management outcomes we assessed, though small sample sizes in the gender-stratified cells limited our ability to estimate our results with precision. Similarly, the relationship between BIGPIC microfinance membership and the HIV screening outcome was stronger in households with lower assets compared to higher assets (Wald *p*-value = 0.05). No statistical differences between household asset status levels were observed for the relationship between microfinance and other health screening and management outcomes.

**Table 3:** Relationship between microfinance group membership, health screening, and disease management, stratified by gender and socioeconomic status among 300 residents of two communities in rural western Kenya, 2018–2019

	Women (*n* = 188)^a^			Men (*n* = 112)^a^			Low wealth^b^			High wealth^b^		
	PR (95% CI)		p	PR (95% CI)		*p*	PR (95% CI)		*p*	PR (95% CI)		*p*
Current NHIF coverage	1.61	(0.73, 3.53)	0.2	1.09	(0.26, 4.64)	0.9	1.60	(0.51, 4.97)	0.4	1.16	(0.42, 3.18)	0.8
*Health screening outcomes*												
HIV screening	1.21	(1.08, 1.35)	0.007	1.26	(1.10, 1.44)	< 0.0001	1.32	(1.16, 1.51)	< 0.0001	1.11	(0.97, 1.25)	0.1
Diabetes screening	3.12	(2.16, 4.50)	< 0.0001	4.00	(2.74, 5.86)	< 0.0001	3.22	(2.17, 4.79)	< 0.0001	3.06	(2.06, 4.54)	< 0.0001
Hypertension screening	1.67	(1.40, 1.98)	< 0.0001	2.67	(2.02, 3.52)	< 0.0001	2.10	(1.68, 2.62)	< 0.0001	1.88	(1.52, 2.33)	< 0.0001
Tuberculosis screening	2.20	(1.02, 4.78)	0.02	12.23	(2.56, 58.46)	0.002	3.19	(1.24, 8.26)	0.02	3.63	(1.26, 10.43)	0.02
Cervical cancer screening	–	–	–	–	–	–	2.38	(0.93, 6.10)	0.07	2.35	(1.04, 5.30)	0.04
*Disease management outcomes, among those diagnosed with HIV, diabetes, or hypertension (n = 32)*												
Medical visit in last 6 months	1.67	(0.98, 2.82)	0.06	1.20	(0.84, 1.72)	0.3	1.37	(0.82, 2.31)	0.2	1.40	(0.57, 3.42)	0.6
Currently taking medication	1.94	(0.95, 3.96)	0.07	1.00	(0.20, 4.95)	1.0	1.65	(0.77, 3.53)	0.2	1.17	(0.23, 5.95)	0.9

^a^Wald *p*-values for interaction terms between gender and microfinance group membership were < 0.05 for the tuberculosis screening outcome only

^b^Wald *p*-values for interaction terms between gender and household wealth asset (dichotomized at median) were < 0.05 for the HIV screening outcome only

Our sensitivity analysis operationalizing microfinance exposure as length of membership instead of as member vs. non-member showed point estimates in the same direction as observed in the primary analyses, though they were not statistically significant (Table 4). Among current microfinance members (*n* = 96), those with longer memberships tended to have more health insurance coverage, higher rates of health screening, and better disease management outcomes. The point estimates for each of these outcomes were above the null (with the exception of the estimate for HIV screening and medical visit within the last 6 months), though these estimates were calculated imprecisely with wide confidence intervals often spanning the null.

**Table 4:** Association between duration of microfinance group membership^a^ and key health screening and disease management outcomes, among current microfinance group members (*n* = 96)

	PR (95% CI)		*p*
Current NHIF coverage	1.38	(0.40, 4.75)	0.6
*Health screening outcomes*			
HIV screening	0.94	(0.88, 1.00)	0.05
Diabetes screening	1.42	(1.01, 1.98)	0.04
Hypertension screening	1.10	(0.95, 1.28)	0.2
Tuberculosis screening	1.09	(0.42, 2.82)	0.9
Cervical cancer screening	1.28	(0.57, 2.90)	0.5
*Disease management outcomes, among those diagnosed with HIV, diabetes, or hypertension*			
Medical visit in last 6 months	0.83	(0.58, 1.19)	0.3
Currently taking medication	2.50	(0.85, 7.31)	0.1

^a^Microfinance group membership duration cutpoint at above/below 12 months

## Discussion

In this study, we found that a microfinance program underpinning the BIGPIC Family intervention in western rural Kenya was strongly associated with increased screening for several key health conditions. Notably, these positive associations were observed for conditions screened for directly by the BIGPIC program (hypertension and diabetes) as well as conditions for which the BIGPIC program does not regularly screen (tuberculosis and cervical cancer). We also found that microfinance members tended to have more HIV screening, better health insurance coverage and disease management outcomes for chronic health conditions, though these findings were not statistically significant.

Our findings align with the generally positive results observed in previous evaluations of the BIGPIC program and of the broader microfinance landscape worldwide. Many microfinance programs, like BIGPIC, have begun integrating health training and healthcare provision into their programs, [34, 35] and have generally found positive health effects, particularly in sexual health and maternal/child health arenas [35–39]. The BIGPIC program has broad objectives to improve the overall health and well-being of Kenyans, but has primarily focused on addressing hypertension and diabetes management. A recent BIGPIC evaluation found participation was associated with increased engagement in care after a hypertension or diabetes diagnosis and consequent reductions in blood pressure [29]. Our study builds on this prior evaluation by examining screening and management of health conditions beyond hypertension and diabetes (i.e. HIV, tuberculosis, cervical cancer) and by contextualizing the results with information on the relationship with health insurance coverage uptake.

Importantly, the most robust results we observed were for health screening outcomes, while the results for health insurance uptake and disease management outcomes were smaller and not statistically significant. There are several plausible explanations for these differences. First, it is possible that these positive relationships exist, but our limited sample size did not support their measurement with adequate precision. Future studies with larger samples would improve precision and clarify the nature of these relationships. Second, education about the benefits of NHIF health insurance coverage is provided through BIGPIC programming; thus, we anticipated uptake of NHIF would be higher than the 14% we observed among the group members. It is possible that it will take more time for an expanding health insurance scheme like the NHIF to gain traction in rural communities with little experience with the program [21]. Until recently, the NHIF had more limited coverage, including limited coverage at some of the facilities in the study area. Thus, the low uptake observed could also be explained by residents in the area rationally choosing not to purchase a product that provided them with limited coverage [40]. NHIF uptake in the study area may naturally expand as knowledge about the expansion of NHIF coverage spreads. Recently, the BIGPIC clinical team has sought to obtain more direct integration with NHIF by incorporating BIGPIC healthcare services as part of the NHIF benefit package. Preliminary findings show new increases in NHIF uptake in the study community when copays are eliminated. Finally, there are likely more barriers for participants to engage in sustained and effective disease management than for health screening at a single point in time. Further, health screening was integrated into the BIGPIC Family program earlier than the disease management components, so participants have had more time to reap the benefits from screening. Programmatic focus on better integrating treatment delivery into BIGPIC Family and in reducing financial and logistical barriers to treatment is ongoing.

Importantly, our findings do not estimate the isolated impact of exposure to microfinance, but rather exposure to the comprehensive suite of interventions the BIGPIC Family program delivers through a microfinance platform. Thus, our ability to understand the mechanisms through which the program operates is limited. Future evaluations should concentrate on understanding the relative impact of each component of the intervention and identifying the optimal set of interventions to produce the largest health impact.

There are several aspects of the study design that warrant cautious interpretation of our findings. First, the cross-sectional design prevented us from establishing temporal relationships between the exposure and the outcomes with certainty. Although we attempted to minimize this concern by asking many of our survey questions in reference to specific time periods, reverse causal interpretations of our findings are still plausible. Second, our health screening and disease management outcomes were self-reported by participants. Social desirability bias may have influenced our results. Finally, though we controlled for several potential confounders in our analyses, unmeasured confounding may still have influenced our estimates. We examined the potential magnitude of this issue with a sensitivity analysis using an alternate microfinance exposure definition, membership duration. This analysis produced similar findings to our main analyses, providing some reassurance that our findings are robust to bias from unmeasured confounding. Future studies with longitudinal follow-up, matching on key sociodemographic characteristics, randomized exposures, and/or healthcare outcomes extracted from medical records will be able to provide clearer insights into the relationships we observed.

## Conclusions

Our study provides preliminary evidence that microfinance programs, like the one underpinning the BIGPIC Family intervention, may have the capacity to increase screening and disease management for some key diseases. Increased screening can dramatically decrease the public health burden of diseases through earlier detection and engagement in care [41]. Similar programs that address barriers to healthcare at multiple levels should be prioritized for further evaluation. Combined microfinance and healthcare delivery programs can be resource-intensive, but may be made more financially sustainable through expanded health insurance coverage. Ongoing efforts should be made to increase health insurance uptake to sustain novel healthcare delivery models like BIGPIC Family and to realize the potential health benefits coverage may provide.

## Supplementary information

**Additional file 1:** **Supplemental Table 1.** Characteristics of the study population of 300 residents of two communities in rural western Kenya, 2018–2019, stratified by microfinance group and two control group communities.

**Additional file 2.** Survey Instrument.

## Data Availability

The data underlying this study are not publicly available because they contain sensitive health information from participants. The data will be made available by the corresponding author on reasonable request.
