# Supplementary material for: The relationship between a microfinance-based healthcare delivery platform, health insurance coverage, health screenings, and disease management in rural Western Kenya
Source: BMC Health Serv Res. 2020 Sep 14;20:868. doi: 10.1186/s12913-020-05712-6 (PMC7491169; doi:10.1186/s12913-020-05712-6)
Supplement: Supplementary file 1 — Additional file 1: Supplemental Table 1. Characteristics of the study population of 300 residents of two communities in rural western Kenya, 2018–2019, stratified by microfinance group and two control group communities. [file 12913_2020_5712_MOESM1_ESM.docx]

**SUPPLEMENTAL TABLE**

**Supplemental Table 1.** Characteristics of the study population of 300 residents of two communities in rural western Kenya, 2018-2019, stratified by microfinance group and two control group communities.

|  |  | **Control group community** | |  |
| --- | --- | --- | --- | --- |
|  | **Microfinance group***  **N=100** | **Community 1**  **N=100** | **Community 2**  **N=100** | **P**** |
| ***Socio-demographic characteristics*** | | | | |
|  | N (%) | N (%) | N (%) |  |
| **Gender** |  |  |  | 0.9 |
| Male | 19 (19.0) | 47 (47.0) | 46 (46.0) |  |
| Female | 81 (81.0) | 53 (53.0) | 54 (54.0) |  |
| **Age** |  |  |  | 0.5 |
| <20 | 1 (1.0) | 8 (8.0) | 15 (15.0) |  |
| 20-29 | 11 (11.0) | 20 (20.0) | 20 (20.0) |  |
| 30-39 | 32 (32.0) | 17 (17.0) | 19 (19.0) |  |
| 40-49 | 30 (30.0) | 26 (26.0) | 20 (20.0) |  |
| 50+ | 26 (26.0) | 29 (29.0) | 26 (26.0) |  |
| **Marital status** |  |  |  | 0.2 |
| Never married | 1 (1.0) | 18 (18.0) | 26 (26.3) |  |
| Currently married | 84 (84.0) | 74 (74.0) | 61 (61.6) |  |
| Divorced/separated | 15 (15.0) | 8 (8.0) | 12 (12.1) |  |
| Missing | 0 | 0 | 1 |  |
| **Education** |  |  |  | 0.3 |
| None/ Some primary | 31 (31.0) | 33 (33.0) | 28 (28.0) |  |
| Primary | 45 (45.0) | 39 (39.0) | 32 (32.0) |  |
| Secondary | 20 (20.0) | 21 (21.0) | 28 (28.0) |  |
| Post-secondary | 4 (4.0) | 7 (7.0) | 12 (12.0) |  |
| **Work outside home (last 30 days)** |  |  |  | 0.2 |
| Yes | 17 (17.0) | 17 (17.2) | 25 (25.0) |  |
| No | 83 (83.0) | 82 (82.8) | 75 (75.0) |  |
| Missing | 0 | 1 | 0 |  |
| **Household asset quartile***** |  |  |  | 0.01 |
| Q1 | 21 (23.6) | 33 (37.1) | 16 (16.7) |  |
| Q2 | 27 (30.3) | 19 (21.4) | 24 (25.0) |  |
| Q3 | 19 (21.4) | 19 (21.4) | 23 (24.0) |  |
| Q4 | 22 (24.7) | 18 (20.2) | 33 (34.4) |  |
| Missing | 11 | 11 | 4 |  |
| **Current NHIF coverage** |  |  |  | 0.8 |
| Yes | 14 (14.1) | 9 (9.0) | 10 (10.0) |  |
| No | 85 (85.9) | 91 (91.0) | 90 (90.0) |  |
| Missing | 1 | 0 | 0 |  |
|  | Mean (SD) | Mean (SD) | Mean (SD) |  |
| **Household size** | 5.2 (2.37) | 4.7 (2.05) | 5.0 (2.36) | 0.5 |
| *Health screening characteristics* | | | | |
| **HIV screening** |  |  |  | 1.0 |
| Yes | 96 (96.0) | 78 (78.0) | 78 (78.0) |  |
| No | 4 (4.0) | 22 (22.0) | 22 (22.0) |  |
| **Diabetes screening** |  |  |  | 0.2 |
| Yes | 77 (77.0) | 27 (27.0) | 20 (20.0) |  |
| No | 23 (23.0) | 73 (73.0) | 80 (80.0) |  |
| **Hypertension screening** |  |  |  | 0.2 |
| Yes | 96 (96.0) | 52 (52.0) | 43 (43.0) |  |
| No | 4 (4.0) | 48 (48.0) | 57 (57.0) |  |
| **Tuberculosis screening** |  |  |  | 0.4 |
| Yes | 20 (20.0) | 7 (7.0) | 4 (4.0) |  |
| No | 80 (80.0) | 93 (93.0) | 96 (96.0) |  |
| **Cervical cancer screening** |  |  |  | 0.4 |
| Yes | 23 (28.4) | 8 (15.1) | 5 (9.26) |  |
| No | 58 (71.6) | 45 (84.9) | 49 (90.7) |  |
| *Disease management outcomes, among those who report HIV, diabetes, or hypertension diagnoses (n=32)* | | | | |
| **Medical visit in last 6 months** |  |  |  | 0.6 |
| Yes | 10 (90.9) | 5 (55.6) | 8 (66.7) |  |
| No | 1 (9.1) | 4 (44.4) | 4 (33.3) |  |
| **Currently taking medication** |  |  |  | 0.3 |
| Yes | 8 (72.7) | 5 (55.6) | 4 (33.3) |  |
| No | 3 (27.3) | 4 (44.4) | 8 (66.7) |  |

*Member of BIGPIC Family microfinance group for at least 6 months prior to interview

**p-value reported for chi-square test for categorical variables and t-test for continuous variables, comparing the two control group communities. P-values calculated among observations with non-missing values.

***Measured by adding up the self-reported value (at time of purchase) of 20 key items in participant’s household
