# Supplementary material for: The relationship between a microfinance-based healthcare delivery platform, health insurance coverage, health screenings, and disease management in rural Western Kenya
Source: BMC Health Serv Res. 2020 Sep 14;20:868. doi: 10.1186/s12913-020-05712-6 (PMC7491169; doi:10.1186/s12913-020-05712-6)
Supplement: Supplementary file 2 — Additional file 2. Survey Instrument. [file 12913_2020_5712_MOESM2_ESM.docx]

**Survey Instrument**

**Community-Based Survey Evaluating the Impact of the**

**Bridging Income Generation through grouP Integrated Care (BIGPIC) Family Model**

**in Bungoma County, Kenya**

| **META-DATA** | | |
| --- | --- | --- |
| 1 | ID |  |
| 2 | Date |  |
| 3 | Interviewer name |  |
| 4 | Latitude |  |
| 4 | Longitude |  |
| 5 | Time interview started |  |
| 6 | Time interview ended |  |

| **RESPONDENT DEMOGRAPHICS** | | |
| --- | --- | --- |
| 1 | Gender | Male  Female |
| 2 | How old are you? | ___ years |
| 3 | What is your marital status? | Never married  Married  Living together  Divorced/separated  Widowed  Other (specify)___ |
| 4 | What is the highest level of schooling you achieved? | None  Completed primary  Completed secondary  Completed post-secondary |
| 5 | In the last 30 days, have you worked outside the home for pay? | Yes  No🡪 Q6 |
| 6 | In the past month, how much income did you receive from your work? |  |
| 7 | Are you the head of the household? | Yes 🡪END MODULE  No |
| 8 | Relation to household head | Spouse  Child  Other (specify)___ |

| **HOUSEHOLD ROSTER** | | | | | | | | | |
| --- | --- | --- | --- | --- | --- | --- | --- | --- | --- |
| **How many people live in your household, not including yourself?** | **(number indicated here tells how many rosters they will get)** | | | | | | | | |
|  |  |  |  | Age 12+ | | Age 6-13 | Age 0-5 | All | |
| 1. Now I'll ask you some questions about the other people who live in your house. Please tell me the nickname of another person who lives in your house. | 2. What is the relationship between [Name] and yourself? | 3. How old is [Name]? | 4. Is [Name] male or female? | 5. In the last 30 days, did [Name] work outside the home for pay? | 6. In the past month, how much income did [Name] receive from their work? | 7. Is [Name] currently enrolled in school? | 8. For which of the following vaccines has [Name] been vaccinated? | 9. How many days in the past month did [HH member] suffer from any sickness? | 10. How many days in the past month did [HH member] miss from work, housework or school due to illness? |
|  | 1, Husband/partner  2, Son/daughter  3, Son-in-law/daughter-in-law  4, Grandchild  5, Parent  6, Parent-in-law  7, Brother/sister  8, Other relative  9, Adopted/Foster child  10, Not related  11, Self  98, Don't know  99, Refuse | In years | Female  Male | Yes  No 🡪 Skip #5 |  | Yes  No | 1.Polio  2.Hepatitis B, 3.Measles,  4.Rubella,  5.Tetanus 6.Tuberculosis  7.Yellow fever  8. Pertussis  9. Mumps  10. Diphtheria | In days | In days |
| 2. |  |  |  |  |  |  |  |  |  |
| 3. |  |  |  |  |  |  |  |  |  |
| 4. |  |  |  |  |  |  |  |  |  |
| Up to 20 |  |  |  |  |  |  |  |  |  |

**HOUSEHOLD ASSETS (Source: DHS Kenya)**

Which of the following items does your household own?

|  | **Item** | **Answer** |
| --- | --- | --- |
| 1 | Electricity | 1. Yes 2. No 3. Don’t Know 4. Refuse |
| 2 | Radio | 1. Yes  2. No  98 Don’t Know  99 Refuse |
| 3 | Television | 1. Yes  2. No  98 Don’t Know  99 Refuse |
| 4 | Mobile telephone | 1. Yes 2. No 3. Don’t Know 4. Refuse |
| 5 | Non-mobile telephone | 1. Yes  2. No  98 Don’t Know  99 Refuse |
| 6 | Refrigerator | 1. Yes  2. No  98 Don’t Know  99 Refuse |
| 7 | Solar panel | 1. Yes  2. No  98 Don’t Know  99 Refuse |
| 8 | Table | 1. Yes  2. No  98 Don’t Know  99 Refuse |
| 9 | Chair | 1. Yes  2. No  98 Don’t Know  99 Refuse |
| 10 | Sofa | 1. Yes  2. No  98 Don’t Know  99 Refuse |
| 11 | Bed | 1. Yes  2. No  98 Don’t Know  99 Refuse |
| 12 | Cupboard | 1. Yes  2. No  98 Don’t Know  99 Refuse |
| 13 | Clock | 1. Yes  2. No  98 Don’t Know  99 Refuse |
| 14 | Microwave oven | 1. Yes  2. No  98 Don’t Know  99 Refuse |
| 15 | DVD player | 1. Yes  2. No  98 Don’t Know  99 Refuse |
| 16 | Cassette or CD player | 1. Yes  2. No  98 Don’t Know  99 Refuse |

**LIVESTOCK AND LAND OWNERSHIP AND USE**

| **1** | How many of each of the following livestock animals does your household own? |  |
| --- | --- | --- |
|  | **Livestock name** | **Number** (NOTE: If exact number is unknown, please estimate) |
| 1a | Cattle |  |
| 1b | Oxen |  |
| 1c | Sheep |  |
| 1d | Goat |  |
| 1e | Chicken |  |
| 1f | Donkey |  |
| 1g | Others (specify) |  |
|  |  |  |
| 2 | [If total livestock owned >0] What are the livestock used for? | Household consumption  Income generation  Both |
| 3 | Do you own any farm land? | Yes  No🡪 END MODULE |
| 4 | What are the crops planted used for? | Household consumption  Income generation  Both |
| 5 | what type of crops are being grown? |  |

| **HOUSEHOLD INCOME** | | |
| --- | --- | --- |
| 1 | What is the largest contributor to your household income? | 1. Crop farming  2. Livestock farming  3. Fish farming  4. Daily labor/piece work earner in agricultural sector  5. Wage earner in non-agriculture large business (factory operation)  6. Petty business (kiosk, trade in farm produce)  7. Artisan (e.g. welder, blacksmith, craftsman, carpenter)  8. Salaried employment/job  9. Remittances  10. Daily labor/piece work earner in health sector  11. Other (specify) |
| 2 | Does your household regularly receive remittances? | Yes  No🡪 Q4 |
| 3 | Approximately how much was the total value of the remittances your household received in the last 30 days? |  |
| 4 | Does your household receive any income from government support programs? | Yes  No🡪 END MODULE |
| 5 | If yes, which program? (check all that apply) | 1.Cash Transfer for Orphans and Vulnerable Children  2. Older Persons Cash Transfer  3. Hunger Safety Net Program  4. Other (specify) |
| 6 | Approximately how much was the total value of the income from government support programs your household received in the last 30 days? |  |

| **HOUSEHOLD LIVING CONDITIONS** | | **Answers** |
| --- | --- | --- |
| 1 | How many rooms are in your home, not including bathrooms and toilets? |  |
| 2 | What materials are used for the main household dwelling roof? | 1 = Grass/straw,  2 = Iron sheet,  3 = Tiles,  4 = other (specify) ________ |
| 3 | What materials are used for the main household dwelling walls? | 1 = Mud,  2 = Mud and twigs,  3 =Wood,  4 = Iron sheet,  5 = Mud and cement,  6 = Block/ brick,  7 = Raw bricks,  8 = Other (specify) ________ |
| 4 | What materials are used for the main household dwelling floor? | 1 = Mud,  2 =Wood,  3 = Mud and cement,  4= Cement,  5 = Tiles,  6 = Other (specify) ________ |
| 5 | What is the main source of drinking water for members of your household? | 1= Piped water  2= Public tap/standpipe  3= Tube well or borehole  4= Protected well  5= Unprotected well  6= Protected spring  7= Unprotected spring  8= Rainwater  9= Surface water (river/dam/lake/pond/stream/canal/  Irrigation channel)  10= Bottled water  11= Other (please specify) |
| 6 | Where is that water source located? | In own dwelling  In own yard/plot  Elsewhere |
| 7 | Do you do anything to the water to make it safer to drink? | Yes  No 🡪Q9  Don't know |
| 8 | What do you usually do to make the water safer to drink?  Anything else?  Record all mentioned. | 1= Boil  2= Add bleach/chlorine  3= Strain through a cloth  4= Use water filter (ceramic/sand/composite/etc)  5= Solar disinfection  6= Let it stand and settle  7= Cover the water container  8= Other  9= Don't know |
| 9 | What kind of toilet facility do members of your household usually use? | 1= Flush or pour flush toilet  2= Flush to piped sewer system  3= Flush to septic tank  4= Flush to pit latrine  5= Flush to somewhere else  6= Flush, don't know where  7= Pit latrine  8= Ventilated improved pit latrine  9= Pit latrine with slab  10= Pit latrine without slab/open pit  11= Composting toilet  12= Bucket toilet  13= Hanging toilet/hanging latrine  14= No facility/bush/field  15= Other |
| 10 | Do you share this toilet facility with other households? | Yes  No |
| 11 | How many mosquito nets does your household have? | Number of nets ____ |
| 12 | How many people in your household slept under a mosquito net last night? | ___ people |

| **FOOD SECURITY** | | **Answers** |
| --- | --- | --- |
| 1 | In the past month was there ever no food to eat of any kind in your house because of a lack of resources to get food?  *1 month=4 weeks/30 days* | 1. Yes 2. No🡪3 3. Don’t know 4. Refuse |
| 2 | How often did this happen in the past month?   1. *month=4 weeks/30 days* | 1. RARELY (1-2 TIMES) 2. SOMETIMES (3-10 TIMES) 3. OFTEN (MORE THAN 10) 4. Don’t know 5. Refuse |
| 3 | In the past month did you or any household member go to sleep at night hungry because there was not enough food?  *1 month=4 weeks/30 days* | 1. Yes  2. No🡺5  98. Don’t know  99. Refuse |
| 4 | How often did this happen in the past month?  *1 month=4 weeks/30 days* | 1. RARELY (1-2 TIMES) 2. SOMETIMES (3-10 TIMES) 3. OFTEN (MORE THAN 10) 4. Don’t know 5. Refuse |
| 5 | In the past month did you or any household member go a whole day and night without eating anything at all because there was not enough food?  *1 month=4 weeks/30 days* | 1. Yes  2. No🡺 End module  98. Don’t know  99. Refuse |
| 6 | How often did this happen in the past month?  *1 month=4 weeks/30 days* | 1. RARELY (1-2 TIMES) 2. SOMETIMES (3-10 TIMES) 3. OFTEN (MORE THAN 10) 4. Don’t know 5. Refuse |

| **ACCESS TO FINANCIAL CAPITAL AND MEMBERSHIP IN GROUPS** | | | | | | | | | |
| --- | --- | --- | --- | --- | --- | --- | --- | --- | --- |
| **QH-1** | **[All]** In the **past 3 months**, have you been in a group-based savings program (e.g. chamas, AMPATH GISE group, SACCOs, formal microfinance groups, merry-go-round, table banking, etc.)? | | Yes **[go to QH-2]**  No **[Go to Section I]** Don’t Know **[Go to Section I]**  Refused **[Go to Section I]** | | | | | 1  2  - 98  - 99 | |
| **Currently** (in the last 30 days), how many groups were you involved with? | | | | | | | | | |
| **QH-2** | Chama/Merry-go-round | | Number of groups | | | ___ ___ | | | |
| **QH-3** | SACCO | | Number of groups | | | ___ ___ | | | |
| **QH-4** | Formal microfinance group | | Number of groups | | | **___ ___** | | | |
| **QH-5** | Table banking | | Number of groups | | | **___ ___** | | | |
| **QH-6** | AMPATH GISE group | | Number of groups | | | **___ ___** | | | |
| **QH-7** | Other: ________________ | | Number of groups | | | **___ ___** | | | |
| **For each of the groups you are currently involved in** (in the last 30 days), we are now going to ask you some additional questions about up to five most recent microfinance groups | | | | | | | | | |
| **Question** | | **Response** | **Group** | | | | | | |
|  |  |  | **1** | **2** | **3** | | **4** | | **5** |
| **QH-8** | What kind of group is this? --> if at least one of the questions from 2-7 is greater than 1 | Chama-1  SACCO-2  Formal microfinance group-3  Table-banking-4  AMPATH GISE-5  Other-6 |  |  |  | |  | |  |
| **QH-9** | How long have you been in this group? | Years  Months | **__ __**  **__ __** | **__ __**  **__ __** | **__ __**  **__ __** | | **__ __**  **__ __** | | **__ __**  **__ __** |
| **QH-10** | On average, how much money have you deposited into this group per month? | Ksh  Don’t Know  Refused | **_ _ _** _ _  -98  -99 | **_ _ _** _ _  -98  -99 | **_ _ _** _ _  -98  -99 | | **_ _ _** _ _  -98  -99 | | **_ _ _** _ _  -98  -99 |
| **QH-11** | How much money have you borrowed from this group in total? | Ksh  Don’t Know  Refused | **_ _ _** _ _  -98  -99 | **_ _ _** _ _  -98  -99 | **_ _ _** _ _  -98  -99 | | **_ _ _** _ _  -98  -99 | | **_ _ _** _ _  -98  -99 |
|  |  |  |  |  |  | |  | |  |
| **QH-12** | In what areas do you spend the money you earn from these groups, across all groups in which you are currently active? (Check all that apply) | A-School fees  B-Medical expenses  C-Insurance  D-Food  E-Business investments  F-Pay off debts  other |  |  |  | |  | |  |

| **ACCESS TO AGRICULTURAL TRAINING** | | |
| --- | --- | --- |
| 1 | Have you received any agricultural training services in the last 12 months? | 1. Yes 2. No🡪 END MODULE 3. Don’’t know 4. Refuse |
| 2 | How many trainings have you received in the last 12 months? |  |
| *[Repeat Q3-4 for the total number of trainings reported in Q2]* | | |
| 3 | What was the main topic covered in the training? | Best crop production practices  Livestock husbandry practices  Basic business management  Crop marketing  Livestock marketing  Value-addition/processing methods  PICS-Purdue Improved Crop Storage Bags  ALV-African Leafy Vegetables  Nutrition  Others (specify)  Don’t know  refuse |
| 4 | Which organization gave the training or service? | 1=Visit by government agricultural extension agent;  2=Visit by government health extension agent  3=Radio;  4=Local administrative meeting (Baraza);  5=TV;  6=Newspaper;  7=Research organizations;  8=AMPATH;  9=Other farmers;  10=Field days/demonstrations;  11=Exchange visits;  12=Shows;  13=Other (specify)_________  14= don’t know  15= refuse |

| **REPRODUCTIVE HISTORY** | | **ANSWERS** |
| --- | --- | --- |
| *[For female respondents age 15-49]* | |  |
| 1 | How many times have you been pregnant? | _____ pregnancies |
| 2 | How many living children do you have? | _____ children |
| 3 | Would you like to have more children? | 1. Yes 2. No 3. Don’t Know 4. Refuse |
| 4 | Are you pregnant now? | 1. Yes  2. No🡺 End Module  98. Don’t Know  99. Refuse |
| 5 | Are you using birth control now? | 1. Yes  2. No🡺Q7  98. Don’t Know  99. Refuse |
| 6 | What birth control method are you currently using (select all that apply)? 🡪SKIP to end | Female sterilization  Male sterilization  IUD  Injectables  Implants  Pill  Male condom  Female condom  Lactational amen. method  Rhythm method  Withdrawal  Other modern method  Other traditional method |
| 7 | What is your main reason for not using birth control? | You are not in a sexual relationship  You can no longer have children  It is too expensive  You do not know where to get it  You are embarrassed to ask for it  You do not know about or understand birth control  You did not think about it  You are worried about the side effects  A healthcare worker refused to give you birth control  You are opposed to birth control  Other (specify) _______ |

| **HEALTH STATUS AND EXPERIENCE WITH HEALTH SCREENING** | | |
| --- | --- | --- |
| 1 | Have you ever been screened/tested for any of the following health conditions? | 1. HIV 2. Diabetes 3. High blood pressure 4. TB 5. Cancer   98. Don’t Know  99. Refuse |
| 2 | [For each infection/condition marked ‘yes’ in Q1, ask:] Who conducted the screening? | 1.BIGPIC Community Screening 2.Events  3.GISHE group-based care  4.Family medicine physicians at Milo 5.Health Center  6.Medical staff at Milo Health Center  7.Matulo dispensary  8.Webuye Subcounty Hospital  9.Othe  98. Don’t Know  99. Refuse |
| 3 | Has a doctor ever told you you have any of the following health conditions? | 1. HIV 2. Diabetes 3. High blood pressure 4. TB   98. Don’t Know  99. Refuse |
| 4 | [For each condition marked ‘yes’ in Q3, ask:] When were you first diagnosed? | MM/YYYY |
| 5 | [For each condition marked ‘yes’ in Q3, ask:] When was your most recent medical visit to monitor this condition? | MM/YYYY |
| 6 | [For each condition marked ‘yes’ in Q3, ask:] Are you currently taking medication for this condition? | 1. Yes  2. No  98. Don’t Know  99. Refuse |

| NATIONAL HEALTH INSURANCE FUND ACCESS | | **Answers** |
| --- | --- | --- |
| 1 | Have you ever heard of NHIF (National Hospital Insurance Fund)? | 1. Yes  2. No  98. Don’t Know  99. Refuse |
| 2 | Do you have NHIF supa+cover? | 1. Yes🡺 Q4  2. No  98. Don’t Know  99. Refuse |
| 3 | Why do you not have NHIF coverage? 🡪 END MODULE | 1.Lack of finances  2.Unsure how to access  3. See no benefit  4. Other (specify)  98. Don’t Know  99. Refuse |
| 4 | How many times have you used NHIF to cover health expenses of your household members in the past 12 months? |  |
| 5 | When did you enroll in NHIF? | mm/yyyy |
| 6 | Do you still wish to keep your membership with NHIF beyond this year? | 1.Yes  2.No  98. Don’t Know  99. Refuse |
| 7 | If no, why not? | 1.Lack of finances  2.See no benefit  3.NHIF not top priority  4.Rarely medicine in hospital  5.Other (specify)  98. Don’t Know  99. Refuse |
| 8 | Do you make the decisions regarding healthcare for this household? | 1.Yes  2.No  98. Don’t Know  99. Refuse |

| **SEXUAL BEHAVIOR** | | **ANSWERS** |
| --- | --- | --- |
| 1 | During the last 12 months, how many sex partners have you had? | ___ partners |
|  | *For your most recent partner:*  *[ONLY FOR THOSE WITH 1+ PARTNERS FROM Q1]* |  |
| 2 | How would you categorize this partner? | 1. Regular 2. Casual 3. Anonymous   98. Don’t Know  99. Refuse |
| 3 | When did this relationship begin? | MM/YYYY |
| 4 | Is this an ongoing relationship? | 1. Yes🡺 Q7  2. No  98. Don’t Know  99. Refuse |
| 5 | If no, when did this relationship end? | MM/YYYY |
| 6 | Is your partner more than 5 years younger or older than you? | 1. >5 years older  2. >5 years younger  3. About the same age  98. Don’t Know  99. Refuse |
| 7 | Has/did your partner ever had sex with someone else during your relationship with him/her? | 1. Yes  2. No  98. Don’t Know  99. Refuse |
| 8 | Do/did you use a condom with this partner? | 1. Always  2. Most of the time  3. Sometimes  4. Never  98. Don’t Know  99. Refuse |
| 9 | Did you use a condom the last time you had sex with this partner? | 1. Yes  2. No  98. Don’t Know  99. Refuse |
